# Assessing mathematics anxiety with the AMAS: Measurement invariance across four countries

**DOI:** 10.1371/journal.pone.0352174

**Published:** 2026-07-08

**Authors:** Serena Rossi, Krzysztof Cipora, Alice Masi, Iro Xenidou-Dervou

**Affiliations:** 1 Centre for Mathematical Cognition, Loughborough University, Loughborough, United Kingdom; 2 Copernicus Center for Interdisciplinary Studies, Jagiellonian University in Krakow, Krakow, Poland; 3 Department of Developmental and Social Psychology, University of Padova, Padua, Italy; Hangzhou Normal University, CHINA

## Abstract

Mathematics Anxiety (MA) affects people of all ages, influencing academic success and daily life. The Abbreviated Math Anxiety Scale (AMAS) is a widely used self-report questionnaire measuring MA and has been psychometrically evaluated in different languages and age groups. However, its cross-cultural measurement invariance, namely whether AMAS items are perceived the same way by people across different countries and cultures, is unknown. Such measurement invariance is a precondition for meaningful cross-cultural comparisons. This study investigated cross-cultural measurement invariance of the AMAS in UK, German, Italian, and Polish university students, using data from 3610 participants from the “Big AMAS database”. We found evidence of partial invariance; only one item (item 8) was perceived differently across countries. Partial measurement invariance indicates that latent factor means of the AMAS can be meaningfully compared across the four considered countries. However, a notable methodological consideration arose – the measurement invariance results heavily depended on the estimator used. This study emphasises the importance of testing for measurement invariance across groups to enable meaningful cross-group comparisons, and it highlights the critical role of the estimation method selection and its impact on the conclusions of psychometric studies.

## 1 Introduction

Mathematics is one of the most fundamental subjects in educational settings. It is not only crucial at school, but it is also very important for everyday life activities (e.g., shopping and managing one’s own finances), and it predicts quality of life [1,2]. Despite its broad importance, many people from childhood to adulthood face mathematics difficulties [3], which can be caused by a variety of factors including cognitive (e.g., mathematical learning disability or difficulty) or emotional factors, such as mathematics anxiety [4].

### 1.1 Mathematics anxiety

Mathematics anxiety (hereafter: MA) has been defined as “a feeling of tension and anxiety that interferes with the manipulation of numbers and the solving of mathematical problems in ordinary life and academic situations” ([5], p.551), or “a negative affective reaction to situations involving numbers, math, and mathematics calculations” ([6], p.197). Although MA is not currently included in any of the diagnostic manuals of mental health disorders, such as DSM-5 [7], ICD-11 [8], it can have a serious impact on the lives of people who experience it. For instance, MA has a negative effect on mathematics performance [9,10]. Most likely the relationship between MA and mathematics performance is reciprocal: poor performance and repeated failure in mathematics can trigger MA in some individuals/children, and MA, in turn, can lead to further reduction of their mathematics performance, creating a vicious circle [11]. This can lead to avoidance behaviours towards the subject during school years [4], and consequently, a missed opportunity to achieve an appropriate education in mathematics. Later, these young people will probably not choose a mathematics-related degree at university because of their scarce preparation, or because they are fearful of mathematics and related assessments [12,13]. However, beyond mathematics-related university degrees (e.g., engineering, chemistry, etc.), mathematics is also involved in many other subjects, such as geography, arts, etc. [14]. Further, it is important for everyday life activities, and for succeeding in higher education. Thus, it is important to examine MA and improve its measurement in university students who can experience different levels of MA, while pursuing their degree.

### 1.2 MA measurement with the Abbreviated Math Anxiety Scale (AMAS)

Self-report questionnaires are currently the most common method of assessing students’ MA worldwide (but see for instance [15,16] for other measurement approaches). Building on decades of MA measurement with several questionnaires (see [17]), in 2003, Hopko, and colleagues proposed the *Abbreviated Math Anxiety Scale* (*AMAS*), originally tested with adult university students in the US.

Although other questionnaires assessing MA have been proposed over the years, the AMAS is currently one of the most used worldwide. It entails nine items, concerning the fear of being tested in mathematics and the fear of learning new mathematical content (two subscales) [18]. This questionnaire is freely available [18], it is easy to administer because it is short (no more than five minutes to complete) and demonstrates excellent psychometric properties [19]. For these reasons, over the last two decades, the AMAS has been translated and psychometrically evaluated in many different languages and age groups (e.g., [20–24]– see Table 1 for a summary. As shown in Table 1, although a one-factor model (MA) was also tested, most validation studies have found that the AMAS has a two-factor structure corresponding to its two subscales, *Learning Mathematics Anxiety* and *Testing Mathematics Anxiety*, which are correlated. In some cases [21,25,26] one item (Item 5: Homework; See Table 3 for the list of all the items) loaded onto both factors. In addition to the expected factor loading on Testing MA, it also loaded on Learning MA factor (two-factors with item 5 double loading model). This suggests that the considered samples perceived a homework assignment, checked by the teacher the following day, as both an assessment and a learning opportunity. Therefore, for the AMAS questionnaire, there are three models worth testing: 1) one-factor model, 2) two-factor model, and 3) two-factors with item 5 double loading.

**Table 1:** Summary of studies with AMAS translations and adaptations.

Authors, year	Country	Sample	Estimator	Authors’ final model
Hopko et al., 2003 [18]	United States	University students	Not reported	Two-factor
Vahedi & Farrokhi, 2011 [27]	Iran	University students	Not reported	Two-factor
Primi et al., 2014 [24]	Italy	University students High school students	MLM	Two-factor
Cipora et al., 2015 [25]	Poland	University students	ADF/WLS	Two-factor with item 5 double loading
Brown & Sifuentes, 2016 [28]	Mexico	University students	–	EFA: Two-factor
Carey et al., 2017 [29]	UK	Primary and secondary school students	WLSMV	Two-factor
Caviola et al., 2017 [20]	Italy	Primary school students	MLM	Two-factors
Cipora et al., 2018 [21]	Poland	University students; employees	ADF/WLS	Two-factors with item 5 double loading
Schillinger, et al., 2018 [26]	Austria	University students	MLM	Two-factors with item 5 double loading
Sadiković et al., 2018 [30]	Serbia	High school students	ML	Bifactor: One general and two specific factors
Szczygieł, 2019 [31]	Poland	Primary school students	MLR	Two-factors
Milovanović, 2020 [32]	Serbia	Primary school students	WLSMV	Two-factors
Martín-Puga et al., 2022 [33]	Spain	Primary and secondary school students	WLSMV	Two-factors
Primi et al., 2020 [34]	Italy	Primary school students (6–7 years old).	MLM	Two-factors

This is an overview only, that covers wider age range than the one analysed in this paper. Some of these studies used some modified items.

MLM: mean-adjusted maximum likelihood; ADF: asymptotically distribution-free; WLSMV: weighted least square mean and variance adjusted; MLR: maximum likelihood robust. EFA: Exploratory Factor Analysis. WLS: Weighted Least Squares.

Note that the “Authors’ final model” column reports the model that each paper accepted as the best one. The type of estimator used indicates whether the data were treated as categorical (WLSMV) or continuous (ML, MLM, MLR, ADF/WLS)

### 1.3 Cross-country differences in mathematics anxiety (MA)

MA research originated in the US [5,35], initially focusing on adults, but has since expanded to include various age groups and countries (see Table 1), investigating MA also from a cultural perspective (e.g., [36–38]). MA can differ from country to country for several reasons: distinct educational systems, languages, cultures, beliefs and attitudes could affect MA differently. For instance, educational systems across different European countries differ substantially from each other in several aspects, such as the total number of years of education and the structure of primary and secondary schools. Mathematics education differs across countries in terms of the curricula, assessment methods (such as different frequency of assessments and their form: written, oral, “pop quizzes”, etc.), teaching, instruments to support learning, and the number of mathematics lessons during the school week [39]. It is important to consider that anxiety may be socially perceived differently across cultural contexts. Even within relatively close cultures like European ones, research has identified between-country differences in stigma, public attitudes, and help-seeking related to anxiety and other mental disorders, reflecting culturally shaped beliefs about their meaning and legitimacy [40,41]. These differences may influence how individuals interpret and respond to anxiety symptoms. However, there is no clear consensus regarding differences in MA levels across countries; some studies highlight significant differences (e.g., [42]), while others suggest that cultural differences in MA are minimal or negligible (e.g., [43]).

Studies investigating MA and its impact on performance or other individual aspects frequently aim to develop interventions that either reduce anxiety or minimise its negative effects on individuals. However, to translate the results of research on MA from one country to another and potentially develop common approaches to alleviating MA in individuals, cross-cultural studies are necessary. Yet, assessing MA can be challenging and requires careful consideration of methodological issues, which will be explained in the next section.

### 1.4 Potential issues of self-report questionnaires within and between different countries: Measurement invariance

As previously mentioned, MA is currently mostly assessed through self-report questionnaires [17]. While these questionnaires have advantages, such as the ability to collect large amounts of data quickly and with minimal effort, they also have several limitations. For instance, people may provide socially desirable answers instead of honest ones (social desirability bias), or they may show a lack of commitment to fill in the questionnaire seriously by consistently responding “Yes” or “No” regardless of the question (e.g., [44]). Additionally, items (i.e., the questions in the questionnaire) can be unclear and may potentially be interpreted differently by different people [45]. All these issues introduce important psychometric considerations that warrant attention.

When developing a new questionnaire or adapting it for different languages and age groups, researchers should evaluate the psychometric properties of the measurement tool, including its reliability. For this latter, contemporary latent variable approaches, such as McDonald’s omega, are increasingly recommended over traditional indices such as Cronbach’s alpha, given the well documented limitations of the latter [46–48]. Further, this questionnaire should be valid (at least in the sample we are considering), i.e., researchers must establish that it truly reflects the construct that it intends to measure. For instance, Confirmatory Factor Analysis (CFA) is often used to assess the construct validity of a questionnaire, that is, whether the measure’s dimensionality is consistent with the theoretical expectations (e.g., one-factor, two factors, and so on) [49]. In other words, CFA determines whether questionnaire items accurately measure the intended construct (e.g., MA), by evaluating whether they show sufficiently high standardised factor loadings on the specified factor/s (construct/s) [45]. It is worth noting that validation of an instrument is an ongoing process, involving the continuous accumulation of evidence, and is therefore neither absolute nor final [50]. Accordingly, when we use the term “validation”, we refer to the collection of evidence supporting the validity of the instrument.

Additionally, it is essential to assess whether the questionnaire items are perceived consistently across all subgroups within the tested population (e.g., genders), that is to check for measurement invariance [51].

Measurement invariance addresses whether scores from a specific instrument assessing an unobserved theoretical construct (e.g., MA) reflect the same meaning under different conditions, i.e., over two or more populations, for example over different genders, countries or at different time points [45]. Therefore, measurement invariance tests whether the questionnaire measures the same hypothesised construct across the considered groups. As recommended in the literature [51], score comparisons between groups/contexts are meaningful only if there is measurement invariance. If there is no measurement invariance between the considered groups/contexts, their mean scores should not be compared because we cannot be sure whether potential differences represent actual differences in the measured construct [52,53]. Therefore, establishing measurement invariance is fundamental to ensure that the observed mean differences can be attributed to differences in the levels of the measured construct across groups [54,55]. On the contrary, in the case of non-invariance, differences in mean scores may reflect different conceptual perceptions of the construct between the to-be-compared groups rather than actual differences in the construct itself. However, it is unfortunately still common practice to conduct comparisons across groups without first ensuring that the items are interpreted equivalently across groups [55].

Regarding the widely used AMAS questionnaire, most of the previous translations and validations in different languages and age groups have demonstrated measurement invariance across genders (e.g., [20,27,33,34]). This means that AMAS measures the same construct among men/boys and women/girls in each of the different populations for which the questionnaire has been validated. But can the same be said about measurement invariance across countries? Before comparing the level of MA between people from different countries and languages, we need to establish whether the groups perceive the questions that they are presented with in a conceptually similar way.

Recently, some studies investigated the potential difference in MA across different countries using different instruments/questionnaires (e.g., [42,56]). However, they did not test for measurement invariance across groups or countries before making these comparisons. Only a few studies have examined measurement invariance of MA across different countries, and most have found partial invariance [36,57]. Partial invariance indicates that the latent levels of MA can still be compared between countries; however, some items in the questionnaire may not be interpreted in the same way by participants from different groups. To the best of our knowledge, none of these studies have used the AMAS questionnaire to investigate the measurement invariance of MA across countries. Therefore, this study is the first to examine cross-country measurement invariance of MA using AMAS.

### 1.5 The present study

The present study is the first attempt to investigate whether we could achieve measurement invariance in MA assessed with the AMAS in university students across four European countries (UK, Germany, Italy, and Poland). The data were obtained from the “Big AMAS database”, an openly available large-scale database of item-level AMAS scores across several countries and different age groups [58], available at https://osf.io/qys6n/. We chose to include these countries due to (a) the availability of data from university students, and (b) the educational systems in these four countries, while sharing some similarities, vary in terms of total years of schooling, mathematics curricula, teaching methods, and assessment practices. Therefore, we hypothesised that these countries could reflect diverse cultural and educational perspectives within the European continent.

Based on the commonly used cut-off criteria for goodness of fit [59,60], we expected that the two-factor model (e.g., [24,27]) or the two-factor with item 5 double loading model would be the best-fitting one [21,25,26] for measurement invariance testing. With the selected model, we then expected to achieve measurement invariance across these four countries. However, since this is the first study to investigate it using this questionnaire, we could not exclude the possibility of obtaining results that contradict our initial expectations.

In the case of achieving measurement invariance across these four countries, the second aim of the study was to investigate whether they differ in terms of levels of MA. The differences in European mathematics education systems could, over the years, impact differently the students’ tendency to feel anxious toward this discipline. Therefore, we expected university students to experience different levels of MA across the countries involved in the study. It is important to highlight that the comparison of the MA levels between countries could only be conducted if we have obtained (at least partial) measurement invariance.

## 2 Method

This study consisted of a secondary data analysis on data from the “Big AMAS database by Cipora and Caviola, 2022 – [58] preregistered in the Open Science Framework (OSF). Additionally, data and analysis scripts, as well as Supplementary materials are available at OSF (https://osf.io/sm2c3/).

### 2.1 Participants

Participants were students attending universities in the UK, Germany, Italy, and Poland. From a more extended dataset, we selected and included in the study only university students over eighteen years old who had responded to all AMAS items. Participants who did not fully complete the questionnaire were deleted from the dataset, therefore we had no missing data. The total sample consisted of 3610 university students (age: M = 22.29, SD = 4.12). Among them, 458 were attending universities in the UK (age: M = 23.4, SD = 6.68), 947 in Germany (age: M = 23.5, SD = 4.17), 668 in Italy (age: M = 21.60, SD = 2.40), and 1537 in Poland (age: M = 21.53, SD = 3.38). Table 2 shows the percentage of participants in each field of study across the different countries, based on the available data. For a detailed breakdown of datasets included in analyses reported here, see Section 2.3.

**Table 2:** Number of participants in each gender and percentage (%) of university students for each field of study in each country.

Country	Gender	N	LIT%	MED%	PED%	PSYCH%	SOC%	STEM%	N/A%
UK (N = 458)	Men	135	18	5	2	38	16	20	1
	Women	313							
	Other	10							
Germany (N = 947)	Men	262	28	10	17	6	13	27	0.1
	Women	685							
	Other	0							
Italy (N = 668)	Men	311	30	0	2	0	0	34	33
	Women	357							
	Other	0							
Poland (N = 1537)	Men	382	11	0.3	23	41	1	24	0.3
	Women	1145							
	Other	10							

LIT = law, history, philosophy, literacy, art; MED = medicine, dentistry, veterinary, nursing; PED = Pedagogy including studies preparing future elementary school children; PSYCH = psychology; SOC = social sciences (e.g., economics, politics, sociology); STEM = Science, Technology, Engineering and Mathematics; N/A = Not Available. Classification of the areas of study based on Cipora & Caviola (2022) (https://osf.io/qys6n/). Note that rounded percentages are reported in the table.

### 2.2 Materials

#### 2.2.1 AMAS.

The AMAS is a self-report questionnaire comprised of 9 items in which participants indicate how they feel about certain mathematics situations using a 5-point Likert scale (1 = low anxiety; 2 = some anxiety; 3 = moderate anxiety; 4 = quite a bit of anxiety; 5 = high anxiety). It comprises two subscales: 1) The *Mathematics Learning anxiety* subscale, which includes five items, assessing the fear of learning new mathematical contents (range 5–25), 2) The *Mathematics Test anxiety* subscale, with four items, assessing the fear of being evaluated in mathematics (range 4–20). Additionally, a total score is calculated by summing up all the items (range 9–45). Higher scores correspond to higher MA. The items of the original (English) version considered in this study are reported in Table 3.

**Table 3:** Items of the AMAS in the English original version.

Instruction	*Please rate each item in terms of how anxious you would feel during the event specified: 1 means low anxiety, and 5 high anxiety.*
**Item 1**	Having to use the tables in the back of a math book
**Item 2**	Thinking about an upcoming math test one day before
**Item 3**	Watching a teacher work an algebraic equation on the blackboard
**Item 4**	Taking an examination in a math course
**Item 5**	Being given a homework assignment of many difficult problems that is due the next class meeting.
**Item 6**	Listening to a lecture in math class
**Item 7**	Listening to another student explain a math formula
**Item 8**	Being given a “pop” quiz in math class.
**Item 9**	Starting a new chapter in a math book.

For the English sample, we considered datasets collected using the original version of AMAS [18]. For the Italian sample, the version translated and psychometrically evaluated by Primi and colleagues (2014) [24]. For the German, the translation by Dietrich and colleagues (2015) [22], and for the Polish, the version translated and psychometrically evaluated by Cipora and colleagues (2015) [25].

### 2.3 Procedure

Data were collected in either paper and pencil or online formats (depending on the aim of the larger study for which the AMAS data were collected). The English sample comes from Rossi and colleagues (online) (2023) [61] and Cipora et al. (paper and pencil) (not yet published). The German data come from Artemenko and colleagues (online) (2021) [62] and Huber and Artemenko (online) (2021) [63]. The Italian data were taken from Primi et al. not included in published papers (paper and pencil), and Lunardon et al., included in a PhD dissertation (online) (2024) [64]. Finally, the Polish data came from published Cipora et al., (2015) (paper and pencil) [25], Cipora et al., 2018) [21] (online), and Sobkow et al., (2021) [65] (paper and pencil) – see specific description in the related OSF project: https://osf.io/qys6n/. Data collection was approved by every university ethics committee within which each data collection was conducted. Only individuals who provided informed consent took part in each study. Each AMAS dataset was collected as part of a larger study which had different aims and research questions than the present study.

### 2.4 Statistical analyses

First, we obtained the descriptive statistics as well as the reliability (Ordinal α) of the AMAS scores in each subscale (*AMAS Learning* and *AMAS Testing*) separately, for the entire sample and each considered country (Table 4).

**Table 4:** Descriptive statistics of AMAS in the two subscales in the entire sample and in each considered country.

Sample	Variable	N	M	SD	Min.	Max.	Ordinal α
Entire	AMAS Learning	3610	8.44	3.70	5	25	0.86 CI [0.50–0.98]
	AMAS Testing		12.86	4.16	4	20	0.88 CI [0.40–0.99]
UK	AMAS Learning	458	9.76	3.98	5	23	0.86 CI [0.47–0.98]
	AMAS Testing		13.18	3.96	4	20	0.87 CI [0.34–0.99]
Germany	AMAS Learning	947	7.49	3.31	5	25	0.91 CI [0.66–0.99]
	AMAS Testing		11.45	4.15	4	20	0.90 CI [0.49–0.99]
Italy	AMAS Learning	668	9.61	4.27	5	21	0.87 CI [0.53–0.99]
	AMAS Testing		13.63	4.28	4	20	0.91 CI [0.52–0.99]
Poland	AMAS Learning	1537	8.11	3.32	5	25	0.81 CI [0.30–0.98]
	AMAS Testing		13.28	3.97	4	20	0.87 CI [0.36–0.99]

AMAS Total scores are not reported as subsequent analyses revealed that one factor model did not fit the data in any of the countries examined.

**Fig 1:**
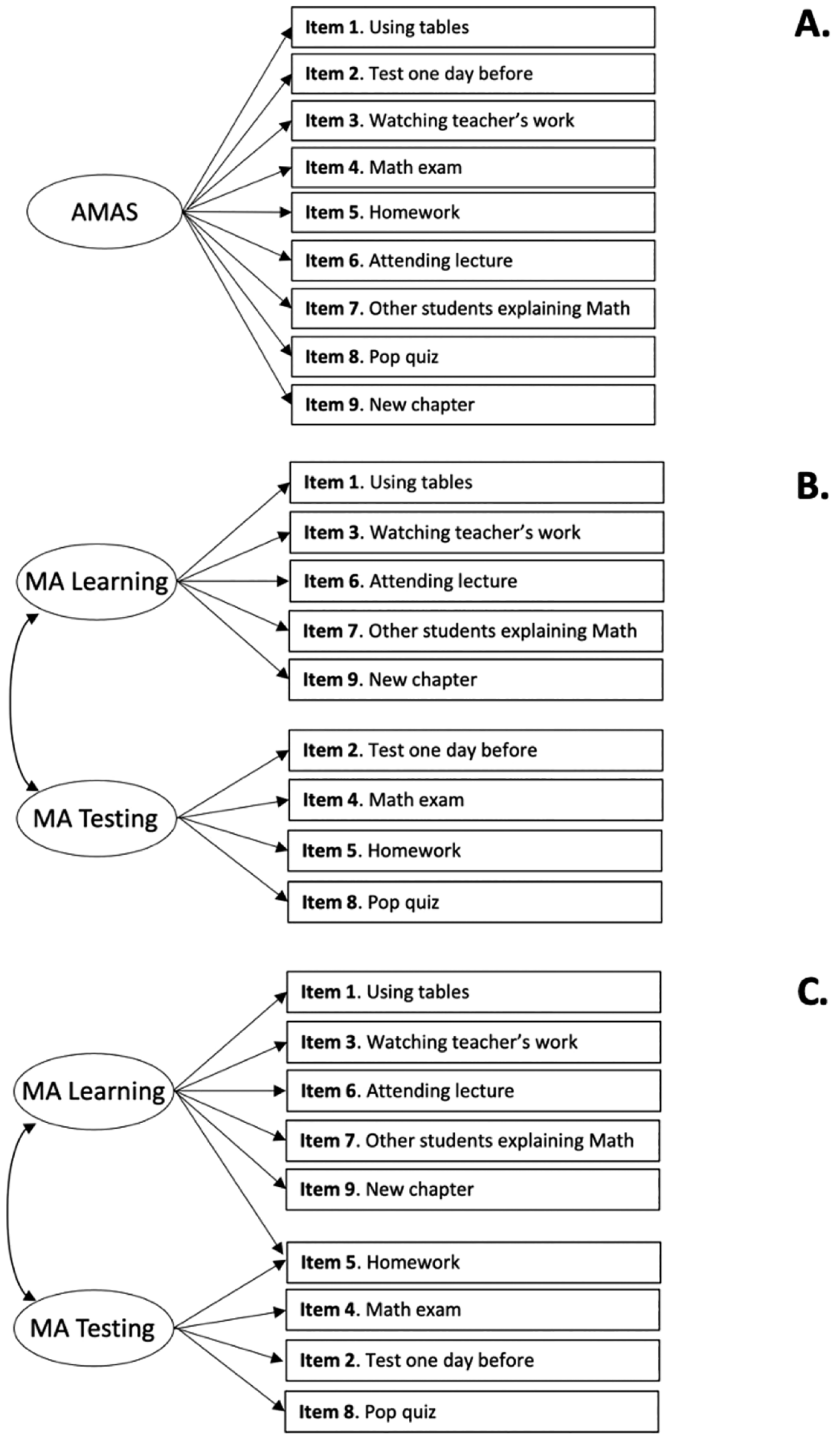
The three AMAS measurement models that we tested using confirmatory factor analysis.

Before testing measurement invariance across the countries, we first examined the factor structure of AMAS (measurement model) that best fit our data through Confirmatory Factor Analysis (CFA). Although we had preregistered this CFA on the entire sample first, this step is not recommended practice in measurement invariance testing (e.g., [66]) and therefore we proceeded with separate CFAs in each country as per step 2 of our preregistration. The first model consisted of the AMAS as a single factor (latent variable) and all 9 items as indicators (observed variables; See Fig 1A). Subsequently, we tested a two-factor model consisting of the two AMAS subscales (*Mathematics Learning anxiety* and *Mathematics Testing anxiety*) as factors (latent variables) correlated with each other, and the respective items as indicators [18] -see Fig 1B. We tested also a third model, which sees item 5 (Homework) loading on both the subscales (two-factors with item 5 double loading; see Fig 1C), as suggested by some studies [21,25,26]. We tested the best fitting model separately for each country to choose the best fitting model and to potentially identify and exclude any countries that did not achieve at least an acceptable fit, as this could otherwise impact the results on measurement invariance (e.g., [54]).

Next, using the model chosen in the previous step, we tested measurement invariance across countries in a step-by-step process: configural, metric, and scalar invariance as per our preregistration. Each step represents a model that is more constrained than the previous one. First, we tested for configural invariance, where factor loadings and items intercepts (thresholds for ordinal data) are set free to vary across groups, testing whether the same factorial structure holds across all groups. If we achieved configural invariance (obtaining good fit indices), we proceeded to testing metric invariance. In this model, factor loadings are assumed to be equal across groups showing that the indices for this model are not significantly worse than those of the configural model. After the metric invariance, we could proceed to testing scalar invariance.

In the scalar invariance, both the factor loadings and item intercepts/thresholds are assumed to be equal across groups. If achieved (fit indices not statistically worse than the metric model), then we could test for strict invariance, where also residual variances are fixed across groups. However, strict invariance is not necessary to achieve measurement invariance. Therefore, if scalar invariance is obtained, then we could compare the scores of AMAS between the considered groups/countries [51,67]. Conversely, if scalar invariance is not achieved, we can test for partial scalar invariance by identifying which constraints, i.e., which items’ intercepts/thresholds, to release. [59,60]. If we achieved at least partial invariance across the four considered countries, we could compare latent factor means of the AMAS across countries, as an exploratory analysis [61].

In the CFA and measurement invariance analyses, model fit was assessed according to commonly used cut-off criteria [59,60], but see [68–70]. We considered the relative fit indices CFI (Comparative Fit Index) and TLI (Tucker-Lewis Index) with thresholds of ≥ 0.90 for acceptable fit (≥ 0.95 for excellent fit); we also considered the residual indices RMSEA (Root Mean Square of Approximation) and the SRMR (Standardized Root Mean Square residual) that would need to be ≤ 0.10 for an acceptable fit (≤ 0.05 for excellent fit). Given the departure from normality observed in some items (see Supplementary materials, S1 Table; https://osf.io/sm2c3/), the data required the use of a robust estimator. In our preregistration, we had initially stated that we would use the Mean-Adjusted Maximum Likelihood (MLM) estimator, based on the assumption that Likert-scale data with five or more points could be treated as continuous variables [71]. However, because Likert-scale data are essentially ordinal, we decided to use a more suitable estimator: the Weighted Least Square Mean and Variance Adjusted (WLSMV), which is specifically designed to be used with ordinal data [71]. With ordinal data and using the WLSMV estimator, only configural and scalar invariance need to be established, while metric invariance is not necessary [72]. This is because, in ordinal models, loadings and thresholds are identified only in combination, making it challenging to separately evaluate metric and scalar invariance [73]. For the comparison of nested models (e.g., configural vs. scalar invariance), we examined the change in the CFI index between models. We accepted models with ΔCFI < .01 [74]. For instance, in the comparison between configural and scalar invariance, if the derived ΔCFI was ≤ .01 then scalar invariance was achieved and we could accept the scalar invariance model. Additionally, we considered the change in SRMR (that needs to be ≤ .03) between the models, as recommended by Chen (2007) [75]. Data analyses were run using R statistical software. For CFA and measurement invariance, we used the R lavaan package [76].

## 3 Results

### 3.1 Descriptive statistics and reliability

Descriptive statistics (means, standard deviation, minimum, maximum) of the AMAS divided into the two subscales, as well as reliability in the entire sample and divided by the considered countries are reported in Table 4. These refer to the two subscales based on the two-factor model proposed originally by Hopko et al., (2003). Therefore, AMAS Learning includes Items 1, 3, 6, 7 and 9, and AMAS Testing includes Items 2, 4 5, and 8, double loading of item 5 is not considered.

### 3.2 CFA in each country

The Confirmatory Factor Analysis (CFA) for the one-factor model demonstrated a poor fit to the data in each country and was rejected based on the absolute goodness of fit indices (See Table 5). The two-factor model showed a slightly better fit, and all the factor loadings were significant (*p* < .001). The third model (two factors and a double loading; see Fig 1C) demonstrated acceptable fit indices and all the factor loadings were significant (*p* < .001). As can be observed in Table 5, the RMSEA value remains borderline for acceptable fit, particularly regarding the upper bound of its confidence interval. However, the RMSEA value can be less trustworthy when working with ordinal data; thus, we do not place strong emphasis on it moving forward, although we report the value for completeness [77]. Given the significant improvement in the indices of the two-factor with item 5 double loading model, we selected this measurement model for the subsequent analyses. Model-based omega values were .77 for Testing MA and .76 for Learning MA, supporting the reliability of the latent constructs without assuming tau-equivalence [78,79].

**Table 5:** Fit indices of the CFA of the models tested in each country.

Sample	Model	CFI	TLI	SRMR	RMSEA [90% CI]	Comparison	ΔCFI	ΔRMSEA	Model accepted
UK	One-factor	.819	.759	.084	.189 [.131 - .161]	–	–	–	–
	Two-factor	.915	.883	.064	.117 [.101 - .133]	–	–	–	–
	Two-factor with item 5 double loading	.934	.906	.058	.109 [.094 - .126]	Two-factor vs. Two-factor with item 5 double loading	.019	.008	Two-factor with item 5 double loading
Germany	One-factor	.817	.756	.219	0.143 [.133 - .154]	–	–	–	–
	Two-factor	.934	.908	.052	.134 [.090 - .112]	–	–	–	–
	Two-factor with item 5 double loading	.951	.929	.043	.118 [.075 - .097]	Two-factor vs. Two-factor with item 5 double loading	.017	.016	Two-factor with item 5 double loading
Italy	One-factor	.818	.758	.088	.202 [.153 - .178]	–	–	–	–
	Two-factor	.940	.917	.050	.118 [.092 - .118]	–	–	–	–
	Two-factor with item 5 double loading	.976	.966	.031	.076 [.042 - .070]	Two-factor vs. Two-factor with item 5 double loading	.036	.042	Two-factor with item 5 double loading
Poland	One-factor	.764	.686	.102	.197 [.157 - .173]	–	–	–	–
	Two-factor	.894	.854	.065	.134 [.108 - .125]	–	–	–	–
	Two-factor with item 5 double loading	.918	.882	.054	.121 [.089 - .106]	Two-factor vs. Two-factor with item 5 double loading	.024	.013	Two-factor with item 5 double loading

**Fig 2:**
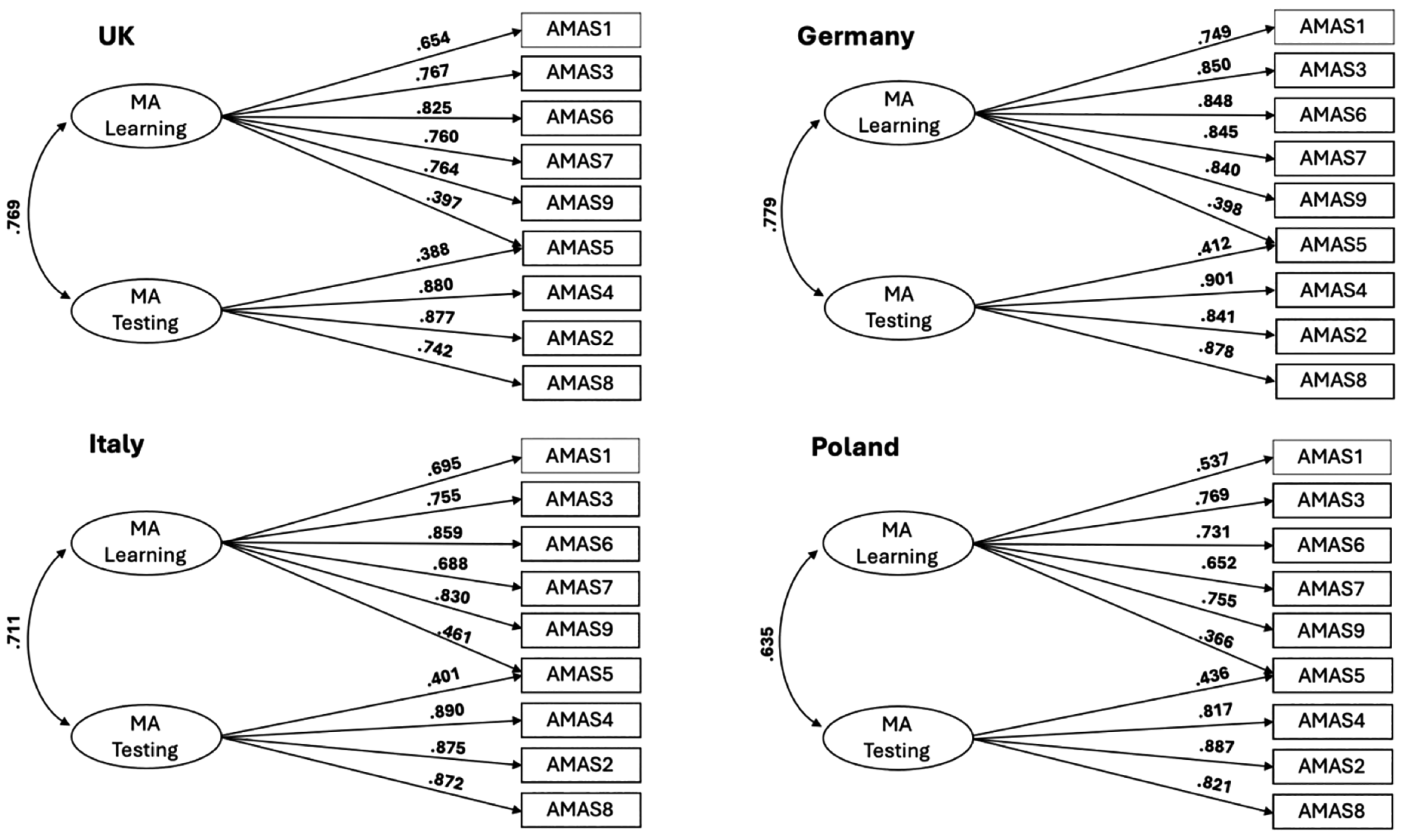
The two-factor with item 5 double loading model with unstandardised parameters achieved for each country.

We then proceeded to test measurement invariance of this model across the four countries. Fig 2 shows the two factor-model with item 5 double loading, with unstandardised parameters achieved for each country.

### 3.3 Measurement invariance across the four countries

The first step was to specify the configural model. This step, compared to the previous one (CFA in each country separately), determines whether the structure is identical across countries (i.e., test of global fit for the structure), providing information at both scale and items levels [66]. It demonstrated an acceptable/good fit to the data (See Table 6, for fit indices). This confirms that the AMAS holds the same two-factor structure (with double loading item 5) in all four considered countries. Achieving configural invariance is a fundamental step for proceeding to test scalar invariance. The lavaan package does not provide robust indices for scalar invariance, only the scaled ones. Therefore, the fit indices reported in Table 6 for configural invariance are the scaled version, to allow comparison with those of scalar invariance.

**Table 6:** Summary of fit indices in the different invariance steps of the two-factor with item 5 double loading model.

Model	CFI	TLI	SRMR	RMSEA	Comparison	ΔCFI	ΔSRMR	Model accepted
Configural	0.979	0.970	0.047	0.090				
Scalar	0.958	0.970	0.052	0.090	Scalar vs Configural	0.021	0.004	Configural
Partial (threshold 1 item 8 free)	0.959	0.970	0.051	0.091	Partial vs Configural	0.020	0.004	Configural
Partial (threshold 1, and 2, item 8 free)	0.961	0.971	0.051	0.088	Partial vs Configural	0.018	0.004	Configural
Partial (threshold 1, 2, and 3 item 8 free)	0.967	0.975	0.051	0.083	Partial vs Configural	0.012	0.004	Configural
Partial (threshold 1, 2, 3 and 4, item 8 free)	0.971	0.978	0.051	0.077	Partial vs. Configural	0.007	0.004	Partial

Scaled indices are reported for all the models due to lack of robust indices in lavaan output for Scalar and Partial invariance.

Scalar invariance (factor loadings and items’ thresholds equal across groups) showed a good fit to the data. The difference in the indices between the configural and the scalar invariance models was above the acceptable cut-off (≤ .010). Even though the ΔSRMR = .005 was within the acceptable cut-off (≤ .030), we could not achieve scalar invariance and tested for partial scalar invariance by releasing some equality constraints across the groups (i.e., setting free to vary certain items’ thresholds across groups). This means that we freed the thresholds suggested by the modification indices from the software, one at a time, to improve the model. We continued this process until, while maintaining acceptable fit indices, the comparison with the configural model indicated that the partial scalar model could be accepted, i.e., it demonstrated fit indices that were not worse than the configural one.

The first modification indices provided suggested that releasing thresholds of item 8 (“pop quiz”) across groups would improve the fit of the model. The AMAS is a Likert-type tool with 5 response categories (1 = low anxiety; 2 = some anxiety; 3 = moderate anxiety; 4 = quite a bit of anxiety; 5 = high anxiety) resulting in 4 thresholds. Threshold represent the cut-off points between categories [45]. For instance, threshold 1 marks the point between selecting “low anxiety” and “some anxiety” (categories 1 and 2). If the threshold is equivalent across groups, it indicates that participants from different countries are likely to interpret and endorse the first two response categories in a similar way [45]. On the contrary, allowing the first threshold of item 8 to differ between the groups means the point at which a person shifts from choosing “low anxiety” to “some anxiety” is not the same between countries.

Since lavaan package requires parameters to be either constrained or freely estimated across all groups, and substantial misfit was observed in the item 8 thresholds for group 2 (Germany) and 3 (Italy), we freed the thresholds, one by one, for item 8 across all groups.

The Partial invariance with threshold 1 of item 8 free to vary across groups, showed a similar fit as found in the scalar invariance. Therefore, we proceeded to release also threshold 2. Although the model showed slightly better indices, ΔCFI between configural and the partial scalar invariance was above the acceptable cut-off. We, therefore, proceeded to release also threshold 3, and ΔCFI was still above the acceptable cut-off. Thus, we also released threshold 4 of item 8. The model showed good fit and this time, both ΔCFI and ΔSRMR between configural and partial scalar invariance models were both within the acceptable cut-off. Table 5 shows the summary of fit indices in different invariance steps.

As a result, we achieved Partial scalar invariance by releasing all the four thresholds of item 8 (“pop quiz”) free to vary between the four considered countries. This means that we can compare at the latent scores of the AMAS across our four countries in a meaningful way [51]. Table 7 shows the unstandardised parameters and standard errors (factors loadings and the thresholds of item 8 of the AMAS across the four countries.

**Table 7:** Partial scalar invariance unstandardised parameters and standard errors of the two-factor with item 5 double loading model.

Item	Loadings Estimate	SE	Constraint	Threshold1	SE_1_	Threshold2	SE_2_	Threshold 3	SE_3_	Threshold 4	SE_4_	Constraint
AMAS2 (Test)	0.885	0.013	Equal	−1.175	0.055	−0.481	0.042	0.121	0.040	0.823	0.049	Equal
AMAS4 (Test)	0.880	0.013	Equal	−1.526	0.063	−0.784	0.045	−0.209	0.040	0.490	0.044	Equal
AMAS5 (Test)	0.434	0.023	Equal	−1.035	0.047	−0.294	0.037	0.394	0.038	1.167	0.050	Equal
AMAS1 (Learn)	0.682	0.022	Equal	0.083	0.035	0.760	0.041	1.367	0.055	2.006	0.082	Equal
AMAS3 (Learn)	0.778	0.021	Equal	−0.096	0.038	0.540	0.039	1.110	0.050	1.692	0.070	Equal
AMAS6 (Learn)	0.854	0.015	Equal	−0.004	0.041	0.716	0.044	1.289	0.057	1.788	0.073	Equal
AMAS7 (Learn)	0.717	0.022	Equal	−0.008	0.035	0.706	0.040	1.250	0.052	1.797	0.073	Equal
AMAS9 (Learn)	0.815	0.017	Equal	−0.125	0.040	0.564	0.041	1.167	0.052	1.776	0.070	Equal
AMAS5 (Learn)	0.416	0.023	Equal	−1.035	0.047	−0.294	0.037	0.394	0.038	1.167	0.050	Equal
AMAS8 (Test)	0.871	0.017	Equal									
		**Country**		**Threshold1**	**SE**_**1**_	**Threshold2**	**SE**_**2**_	**Threshold 3**	**SE**_**3**_	**Threshold 4**	**SE**_**4**_	**Constraint**
		UK		−1.496	0.074	−0.994	0.074	−0.488	0.051	0.030	0.049	Different
		Germany		−1.751	0.093	−1.114	0.063	−0.555	0.049	0.153	0.052	Different
		Italy		−1.077	0.095	−0.316	0.068	0.330	0.068	0.964	0.099	Different
		Poland		−1.657	0.092	−0.887	0.059	−0.239	0.046	0.357	0.049	Different

SE = Standard Error.

**Fig 3:**
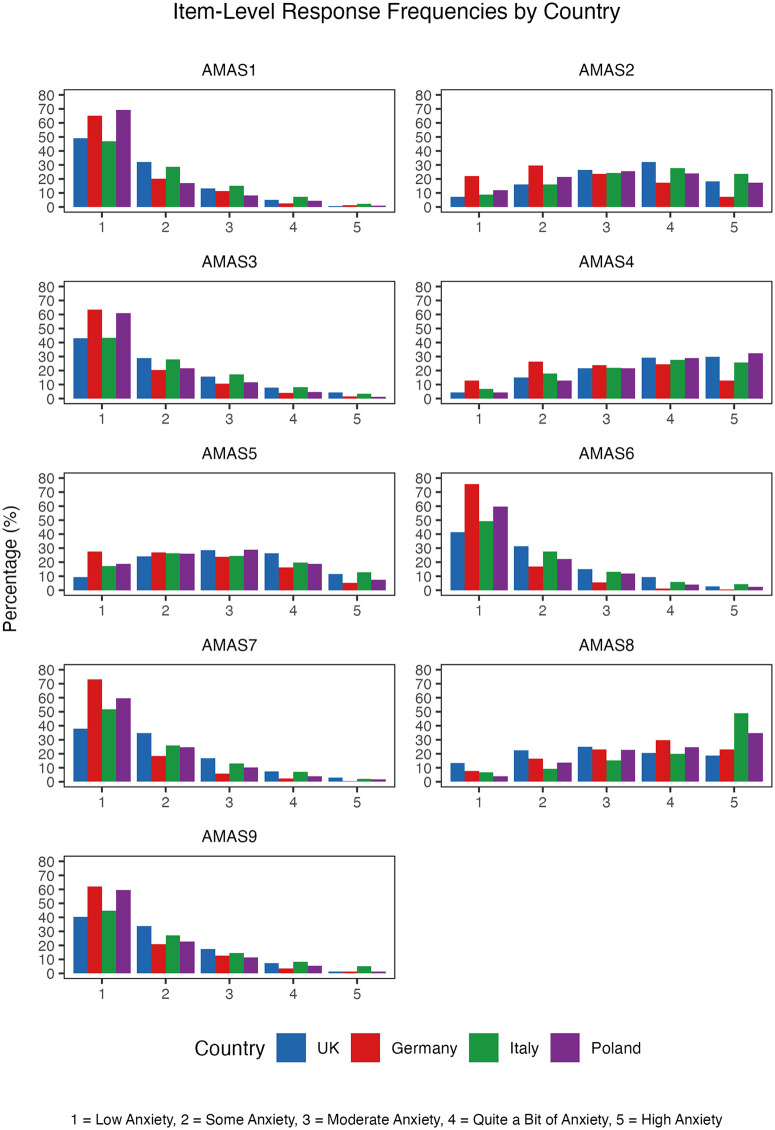
Item-level response frequencies for each AMAS item by country.

Fig 3 presents item-level response frequencies for each AMAS item across the four countries, offering descriptive context for the partial scalar invariance findings. With the exception of items 8 (AMAS8), response distributions were broadly consistent across countries, displaying a right-skewed profile indicative of lower anxiety endorsement. Item 8 showed a markedly different pattern, with Italian respondents endorsing higher anxiety responses at a substantially greater rate than all other countries, providing visual support for the non-invariant thresholds identified for this item.

### 3.4 MA level comparisons between countries

As preregistered, we proceeded to test differences in MA in the two subscales between the four countries as an exploratory analysis. Since we achieved partial scalar invariance and not full scalar invariance, differences in latent factor means across countries were examined. No significant differences were found across countries for Testing MA, F(3,3606) = 0.07, *p* = .977, or Learning MA, F(3,3606) = 0.15, *p* = .931.

### 3.5 Measurement invariance considering the AMAS items as continuous variables (using the MLM estimator)

Given the inconsistency of the use of different estimators across the literature, we also briefly report the results (see full description in the OSF-https://osf.io/sm2c3/) we obtained using the MLM estimator. As mentioned earlier, we had initially preregistered to use the Mean-Adjusted Maximum Likelihood (MLM) estimator for the measurement invariance analyses, which is used when data are considered continuous and when some items show departure from normality [61]. Using the MLM, we still found that the best model to be the one with two factors and item 5 with double loading. However, when we tested for measurement invariance we only obtained configural invariance, CFI = .958, TLI = .940, RMSEA = .081 [90% CI = .075, .087], SRMR = .043, but not the metric one, CFI = .946, TLI = .938, RMSEA = .082 [90% CI = .077, .088], SRMR = .062 across all the four countries, (ΔCFI = .012 above the acceptable cut-off, ≤ .010). Metric invariance is a fundamental step to achieve with continuous variables, to be able to test the third step of measurement invariance (e.g., [45]). This forced us to stop the process and conclude that we did not achieve measurement invariance in the AMAS across all four countries.

## 4 Discussion

This study assessed whether the AMAS questionnaire, one of the most commonly used self-report instruments for assessing Mathematics Anxiety (MA) worldwide, achieves measurement invariance among university students across four countries: the UK, Germany, Italy, and Poland. We used data from an openly available large database containing AMAS scores across different countries, languages, and age groups (“Big AMAS database” Cipora & Caviola, https://osf.io/qys6n/). Full (scalar) measurement invariance could not be established across the four countries examined, but we achieved partial scalar invariance, with only one item (Item 8: *“Being given a pop quiz in math class”*) interpreted differently across the four groups. This partial invariance suggests that AMAS scores can still be meaningfully compared at the latent level across these countries [51], therefore supporting the use of AMAS in cross-country studies, at least among the four countries considered in the present study. Importantly, the measurement invariance results differed when the AMAS item responses were treated as continuous variables, as done in several previous studies, rather than as ordinal, which more accurately represents the nature of Likert-scale data. These findings carry both theoretical and methodological implications for MA research, which are further discussed below.

### 4.1 Measuring MA with AMAS

First, we wanted to identify which of the AMAS factor models suggested in the literature better explained our data, by examining the factor structure of the AMAS questionnaire each country. We examined whether all the items included in the AMAS assess one latent theoretical factor (MA), or two separate ones (*Mathematics Learning anxiety* and *Mathematics Testing anxiety*). We also investigated whether item 5 (“*Being given a homework assignment of many difficult problems that is due the next class meeting.*”) loads on both the Learning and Testing MA components, as found in some previous studies (e.g., [21,25]). The CFA conducted in each country indicated that this third model structure provided the best fit for our data. This finding partly confirms the original two-factor structure of the AMAS postulated by Hopko and colleagues [18], but it also indicates a double loading of item 5 on both factors, as previously observed by [21,25] in a substantial portion of Polish sample, which is also included in the present study. This suggests that our large international sample perceived doing a homework assignment, which will be checked by the teacher the day after, not only as a testing experience but also as a learning opportunity. This also indicates that this model is a plausible representation of the data in each of the considered countries and provides valuable insight into the underlying structure of MA. However, this step could not determine whether the structure of the AMAS was identical or not across all four countries, i.e., a test of global fit for the structure. To address this, we conducted a multi-group analysis to test measurement invariance across the countries, obtaining information at both scale and item levels [66].

### 4.2 Is there measurement invariance across countries?

Measurement invariance across groups in a specific instrument is a prerequisite for meaningful comparison of at least latent factors means between those groups [51]. However, unfortunately, testing for measurement invariance before conducting the comparison remains an uncommon practice for psychology research, increasing the risk of misleading conclusions [55]. The present study was the first one which tested the measurement invariance of the AMAS questionnaire across four different countries. It showed that we could achieve the first step of the invariance, configural invariance, which means that the AMAS model had the same latent structure across these four countries. Configural invariance means that the items in the questionnaire (e.g., “Thinking about an upcoming math test one day before*”*) can be used (are good indicators) to measure the theoretical construct, e.g., MA Testing factor, in each group [66]. We then tested for scalar invariance. In the full scalar invariance model, both the factor loadings and item intercepts/thresholds are assumed to be equal across groups. Equal factor loadings indicate that a one-unit increase in an item (e.g., selecting “3” instead of “2”) corresponds to the same increase in the latent construct score across all countries [66]. Having also equal items’ intercepts/thresholds implies that items have the same point of origin across groups [51,80]. This implies that participants with the same level of the latent construct should have equal expected item responses [81]. Under these conditions, the AMAS scores can be meaningfully compared across countries [51].

Because the AMAS items use a Likert-type scale (ordinal), we treated the responses as ordinal variables and applied the WLSMV (Weighted Least Square Mean and Variance adjusted) estimator. Therefore, in the scalar invariance model, we constrained item thresholds, which are cut-off points between response categories. For instance, threshold 1 is the cut-off point between selecting “low anxiety” and “some anxiety. If the threshold is equivalent across groups, it indicates that participants from different countries are likely to interpret and endorse the first two response categories in a similar way [45].

In our study, the AMAS did not achieve full scalar invariance; therefore, we tested for partial scalar invariance. Based on the modification indices provided by the software, we sequentially released all four thresholds of item 8. This partial invariance model could be accepted. Thus, we concluded that partial scalar invariance was achieved, allowing for meaningful comparison of latent factor means of the AMAS across the four countries.

This partial invariance indicates that item 8 of the AMAS was the only item not interpreted in the same way across the four countries. This item refers to the participant’s level of anxiety in response to *“Being given a pop quiz in a math class”*. Differences in how certain items are perceived across countries can have multiple explanations. One possible factor is the language of administration, which varied among the four countries (i.e., English, German, Italian, and Polish). It is possible that in the questionnaire’s validation process in each country, the translation of some of the items, such as item 8, may have slightly altered their meaning, thereby affecting how respondents interpreted them (e.g., [82]). Another plausible explanation can be attributed to cultural differences between countries, that is educational systems could have also influenced differently the development of MA perceptions of specific items. On this latter note, we can also assume that some situations described in the items and in which participants had to imagine themselves being in and report their level of anxiety, can be seen differently based on previous and current experiences in educational and cultural settings. This may be particularly true for item 8 (‘pop’ quiz), which has the highest mean score among AMAS items across the entire Big AMAS database. This is not surprising, as we can hypothesise a “surprise” mathematics test is an anxiety-inducing situation for most people. However, how we perceive this item and respond to it may depend on our linguistic and educational experiences. In American English, a “pop quiz” refers to “a short test that a teacher gives without any warning, to check whether students have been studying or retained taught content” [83]. However, this term is not commonly used in the UK, where it might even be confused with the relatively relaxed format of “pub quiz”. Additionally, previous studies have shown that measurement invariance may be distorted depending on whether items refer to an individual’s lived experiences or hypothetical scenarios [84]. The use of pop quizzes varies across educational systems. For instance, they are sometimes used in Polish schools but are rare in UK schools. Thus, Polish participants may have responded by recalling actual pop quizzes experiences, while UK participants likely imagined a hypothetical scenario [81].

Achieving partial scalar invariance means that we could meaningfully compare AMAS subscales latent means across the four countries, which revealed non-significant cross-country differences in the latent level of MA. Although the main aim of the study was to test measurement invariance in the AMAS, rather than examine differences in MA level across countries, future research should further investigate factors (e.g., social, educational, and economic) that may contribute to potential cross-national differences in MA. While we found at least partial invariance in the AMAS across the countries, this study also raised a methodological issue regarding how Likert-scale data should be treated and analysed.

### 4.3 Methodological considerations in the use of different estimators

In our measurement invariance analysis we used an estimator suitable for ordinal data (WLSMV) [71,85], as the AMAS scale uses a Likert-type format [71]. However, initially, we had preregistered a different estimator (MLM for continuous variables) as it has been commonly accepted that Likert-scale variables with 5 or more response categories can be analysed using an estimator for continuous variables [71]. This approach has also been adopted by several studies, including various validations of the AMAS across different languages and age groups (e.g., [24,26,86]). However, the results in our study differed when using the MLM estimator compared to the WLSMV estimator (no measurement invariance vs. partial invariance).

The differing results obtained from the two analytical approaches, despite using the same dataset, raise an important methodological concern that applies not only to research on MA but also to many other studies that rely on Likert-type scales. A key issue is the inconsistent way researchers handle the statistical analyses of those data, particularly concerning the choice of estimator used in factor analysis and related models (e.g., measurement invariance). The general idea in psychology is that a Likert-type scale, even though ordinal/discrete in its nature [85], reflects an underlying continuum. Therefore, the data are assumed to behave similarly to continuous data [87–89], justifying the use of maximum likelihood (ML) estimators (e.g., [71]), which are older and relatively simpler than ones suited for ordinal data. However, this assumption has led to widespread confusion, as researchers often choose either continuous or ordinal estimators without providing a clear justification. Our review of the literature specifically related to the AMAS questionnaire, as shown in Table 1, confirms that many studies adopt the continuous approach without reporting additional characteristics that might support their choice. We initially followed the same path by preregistering the use of MLM. The discrepancy found in our study across the two approaches highlights that the choice of estimator is not just a technical detail (sometimes not even reported in published papers), but something that can substantially influence the findings and conclusions of a study.

This situation points to an urgent need for clearer guidelines on when (and if) Likert-type data can be treated as continuous. Although the development and selection of appropriate estimation methods are key areas of focus in quantitative research (e.g., [71,90]), it is essential that future work, particularly by methodological experts, focuses on developing a clearer framework to help researchers make more informed decisions in this area.

Until such guidance is available researchers should proceed with caution. Where possible, they should use statistical methods that align with the true nature of the data, which, in the case of Likert-type scales, is ordinal. By contrast, the MLM estimator is more appropriate when the data are truly continuous, for instance when using sliders or visual analog scales, which can be readily implemented in computerised measurement [87–89]. It is also advisable to check whether results remain consistent when analysed using both the ordinal and the continuous estimator, and report any differences. Full transparency in explaining and justifying all analytical choices is crucial, as is making data and code available to allow others to re-analyse the results using different methods, if needed.

Improving these practices also requires more awareness in the research community. Journals and reviewers should encourage authors to think more critically about the assumptions behind their statistical models, and statistical training programs should teach about the implications of treating ordinal data as continuous. By adopting more consistent and careful approaches, future studies can produce more rigorous and comparable results, not only in the field of MA but across any area of research that uses Likert-type scales.

### 4.4 Limitations of the study

Beyond several strengths, this study also has some limitations. First, we considered only four countries and only within the European continent (educated, industrialised and democratic countries), because these were the available data involving university students in the “Big AMAS database”. This open online database [58] is constantly expanding with AMAS scores from different countries and age groups. It would be relevant for researchers worldwide to contribute to this dataset and create similar resources for other popular instruments. Future studies could assess the questionnaire’s measurement invariance across more countries, in particular going beyond the Global North, including different age groups. The shared analyses script of this paper can be used by researchers for this purpose (see OSF: https://osf.io/sm2c3/). This would be useful to improve our knowledge base and understanding of the MA phenomenon in a global manner.

Another potential limitation of the study is that it considers data collected both in person and online, sometimes even within the same country. Although Cipora and colleagues [21] found no differences in the psychometric properties of the AMAS between online or paper and paper-and-pencil administration formats, future studies should consider AMAS data collected in either one modality or both in a more systematic way and control for it to avoid potential differences.

In addition, we did not account for potential multilevel clustering (e.g., participants nested within study programmes or university), as relevant grouping information was not available in the “Big AMAS database”. While a lot of the data was collected individually online, some clustering effects may still be present and should be considered in future studies.

For some of the datasets included the students’ field of study was not available (see Table 2), which limited our ability to analyse this aspect. Future studies should take this variable into account, as it can be hypothesised that questions related to MA may be perceived differently by students enrolled in maths-related versus non-maths-related university courses.

Furthermore, we used the “lavaan” package in R software to perform the analyses because it is an Open Source software and makes our analyses accessible to everyone. However, it requires parameters to be either constrained or freely estimated across all groups, and therefore we released the thresholds of item 8 (one by one) in all 4 countries and not only in some of them. This is a limitation of this study because partial invariance of specific thresholds or countries could still be worthwhile investigating.

Finally, we used the traditional analytical approach to test for measurement invariance. However, there are other methods for investigating parameter invariance such as the alignment method [91] and Bayesian approaches (e.g., [92]). Future studies could apply all these methods to a single dataset to examine the differences, as well as advantages and disadvantages, of each approach.

### 4.5 Conclusions

This study demonstrated that partial measurement invariance of the AMAS questionnaire could be established among university students across four countries (UK, Germany, Italy, and Poland), allowing for meaningful comparison of latent factor means between these countries.

It is important not to take for granted that items of a questionnaire, in this case the AMAS, are perceived in a similar conceptual way between people from different countries with different cultures, education and societal systems, as reported in a recent study [55]. Just as it is necessary to test for measurement invariance across genders in a specific questionnaire before conducting analyses with any forms of gender comparisons [93], this study highlights the importance of testing and confirming measurement invariance in questionnaires before comparing their scores between countries. This is crucial even when a questionnaire, such as the AMAS, is standardised and psychometrically evaluated across different languages and cultural groups. More broadly, for other constructs and theories, establishing measurement invariance should be the initial step to ensure meaningful and valid comparisons between groups (e.g., [51,54]). Therefore, future studies in the social and psychological fields should adopt this as a standard practice, especially when assessing constructs whose scores can be differently influenced by participants’ group memberships. This ensures the ability to make inferences and generalisations of results across groups, and, in our case, for MA across countries.

Finally, given the differing results obtained with the two different estimators (for ordinal vs. continuous data), the study raises broader questions about the extent to which entire study conclusions depend on analytical choices, particularly the selection of specific (and often technical) methods commonly used in similar research. More specifically, it highlights the critical need for guidance from methodological experts regarding the conditions under which Likert-scale points can be treated as continuous data, if at all. Such guidelines would help researchers establish a standard approach, promoting consistency and comparability in future studies.
